# Trends in Second‐Line Initiations and Treatment Outcomes Across Age and Frailty Groups in People With Type 2 Diabetes: UK Population‐Based Study, 2019–2024

**DOI:** 10.1111/dom.70960

**Published:** 2026-06-04

**Authors:** M. M. Dinsdale, K. G. Young, P. Cardoso, L. M. Güdemann, T. T. Jansz, A. P. McGovern, A. G. Jones, E. R. Pearson, A. T. Hattersley, T. J. McKinley, B. M. Shields, J. M. Dennis

**Affiliations:** ^1^ Clinical and Biomedical Sciences University of Exeter Medical School Exeter UK; ^2^ Division of Diabetes, Endocrinology and Reproductive Medicine Ninewells Hospital and Medical School, University of Dundee Dundee UK

**Keywords:** pharmaco‐epidemiology, primary care, SGLT2 inhibitor, type 2 diabetes

## Abstract

**Aims:**

To assess contemporary UK trends in second‐line treatment initiations and outcomes across age‐and‐frailty groups in people with type 2 diabetes (T2D).

**Materials and Methods:**

We studied 117 046 UK adults with T2D initiating second‐line therapy between 2019–2024 (Clinical Practice Research Datalink). Outcomes were assessed in three groups: age ≤ 70 years (*n* = 84 589 [72%]); non‐frail > 70 years (*n* = 18 933 [16%]); frail > 70 years (*n* = 13 524 [12%]). Annual prescribing trends by drug class were described and we evaluated annual changes in 12‐month HbA_1c_, weight and severe diabetes‐related complication outcomes.

**Results:**

SGLT2‐inhibitor use increased from 26% of overall second‐line initiations in 2019 to 63% in 2024, with the greatest increase among frail adults > 70 (6% in 2019 vs. 60% in 2024). In adults ≤ 70, mean 12‐month HbA_1c_ response improved from −11.3 mmol/mol (95% CI: −11.6 to −11.0) in 2019 to −12.9 mmol/mol (95% CI: −13.2 to −12.6) in 2023 and mean weight loss increased from −1.7 kg (95% CI: −1.8 to −1.5) to −2.8 kg (95% CI: −2.9 to −2.6). Incidence of short‐term severe diabetes‐related complications remained similar: 9.9 (95% CI: 8.1 to 11.7) per 1000 person‐years in 2019 versus 8.6 (95% CI: 6.9 to 10.3) in 2022. Similar time trends in treatment outcomes were seen across all age‐and‐frailty based subgroups.

**Conclusions:**

Use of SGLT2‐inhibitors rapidly increased in the UK over 2019–2024, with a 10‐fold increase among older adults with frailty. Over the same period, there were small population‐level benefits for HbA_1c_ and weight, with no evidence of changes in complication risk. Findings support careful use of SGLT2‐inhibitors across the wider population with T2D, including older adults with frailty.

## 1 Introduction

Type 2 diabetes affects over 700 million people [1] and most individuals will require glucose‐lowering therapy in their lifetime to achieve and maintain glycaemic control. Metformin is almost universally recommended as first‐line treatment [2], but for many people there is a lack of guidance on which second‐line therapy to prescribe after metformin and recommendations differ between national and international guidelines [3, 4]. In practice, this lack of clarity means treatment decisions are often shaped by clinician and patient preference, resulting in marked variability by geography and patient characteristics [5, 6, 7, 8, 9].

SGLT2‐inhibitors (SGLT2i) offer benefits beyond glycaemic control, including weight loss and cardiorenal protective effects [10]. Reflecting this evidence, international guidance recommends SGLT2i independently of glycaemic control for people with cardiovascular or chronic kidney disease and to consider them for those at high cardiovascular risk [3, 4, 11]. New UK National Institute for Health and Care Excellence (NICE) guidance extends this to recommend SGLT2i first‐line alongside metformin for all people with type 2 diabetes except those with frailty [12]. Despite this, recent studies across multiple countries including the UK have shown uptake of SGLT2i remains lowest in those most likely to benefit, including those with cardiovascular or kidney disease and in older adults [13, 14, 15, 16, 17]. To date, however, no studies have examined UK type 2 diabetes prescribing trends beyond 2020 or recent trends in population‐level treatment outcomes after initiating therapy [6].

We aimed to describe contemporary prescribing trends for second‐line glucose‐lowering therapies after metformin in the UK between 2019 and 2024, with a particular focus on frail older adults where SGLT2i are prescribed with caution [15]. We further evaluated trends in short‐term clinical outcomes after second‐line initiation, including glycaemic response, weight change, treatment discontinuation and severe diabetes‐related complications.

## 2 Materials and Methods

### 2.1 Study Design and Participants

We conducted a population‐based cohort analysis using data from the UK Clinical Practice Research Datalink (CPRD) Aurum, a primary care database covering approximately 13% of the population in England and broadly representative of the UK population [18]. Primary care records were linked to hospital admission data (Hospital Episode Statistics [HES]), Office for National Statistics (ONS) death registrations and individual‐level 2019 English Index of Multiple Deprivation (IMD), an area‐level measure of relative deprivation across income, employment, education, health, crime, housing/services and living environment [19]. We included adults diagnosed with type 2 diabetes who initiated second‐line glucose‐lowering therapy after initial treatment with metformin between 1 January 2019 and 1 March 2024. Cohort identification followed our previously published protocol [20] (see https://github.com/Exeter‐Diabetes/CPRD‐Codelists for all codelists). HES data were available up to March 2023.

Second‐line medications were categorised by major drug class into DPP4‐inhibitors (DPP4i), GLP‐1 receptor agonists (GLP‐1RA), SGLT2i, sulphonylureas and other (combined acarbose, glinides, thiazolidinediones and insulin, as prescribing numbers were low for each of these drug classes).

#### 2.1.1 Covariates

We extracted baseline participant characteristics at the time of second‐line treatment initiation including age, sex, duration of diabetes, ethnicity (categorised according to major UK census categories: White, South Asian, Black, Mixed, Other), social deprivation (IMD quintile where 1 indicates least deprived and 5 most deprived), BMI (kg/m^2^), weight (kg), HbA_1c_ (mmol/mol), cardiovascular disease (CVD: myocardial infarction, stroke, revascularisation, ischaemic heart disease, angina, peripheral arterial disease, transient ischaemic attack), chronic kidney disease (CKD) stages 3–5, heart failure, hypoglycaemia, hyperglycaemia, lower limb amputation and severe retinopathy (vitreous haemorrhage, retinal photocoagulation).

#### 2.1.2 Age and Frailty Subgroups

Temporal prescribing trends and clinical outcomes were assessed within three subgroups defined by age and frailty status at second‐line therapy initiation: age ≤ 70 years, non‐frail > 70 years and frail > 70 years. Frailty was measured using the electronic frailty index (eFI) [21], a validated cumulative deficit model based on the presence of 36 health deficits recorded in primary care. Each individual's eFI score was calculated as the proportion of recorded deficits prior to second‐line therapy initiation. Individuals were classified as frail (moderate/severe frailty, eFI > 0.24) or non‐frail (fit/mild frailty, eFI ≤ 0.24) [22].

### 2.2 Study Outcomes

We examined calendar‐year trends (2019–2024) in initiation of second‐line glucose‐lowering therapies, as well as trends in short‐term clinical outcomes after initiating therapy, including HbA_1c_ (glycaemic response), weight change, treatment discontinuation and severe diabetes‐related complications.

#### 2.2.1 Glycaemic Response and Weight Change

We defined glycaemic response and weight change as the absolute change from baseline to 12 months following initiation of second‐line therapy. Baseline HbA_1c_ was defined as the closest measurement within the 6 months prior to the drug start date. Baseline weight was defined as the closest measurement within the 2 years prior to the drug start date. HbA_1c_ and weight at 12‐months were defined as the closest recorded value to 12 months post‐treatment initiation, within a window of 3 to 15 months, with no addition or cessation of other glucose‐lowering therapies and continued prescription of the drug of interest. Outcomes were evaluated by calendar year of drug initiation over 2019–2023, allowing for sufficient follow‐up to assess 12‐month treatment effects.

#### 2.2.2 Treatment Discontinuation

We defined treatment discontinuation as cessation of the drug of interest within 12 months of initiation. Patients were required to have 3 months of follow‐up time after their last prescription to confirm that the drug was discontinued [23]. We included patients who initiated therapy between 2019–2022, allowing sufficient follow up time to accrue (12 months for potential treatment duration and an additional 3 months of follow‐up).

#### 2.2.3 Diabetes‐Related Complications

Severe diabetes‐related complications were assessed as a composite outcome, measured as the first occurrence of any event after drug initiation, defined according to recent UK Prospective Diabetes Study (UKPDS) criteria [24]: sudden death; death due to hyperglycaemia or hypoglycaemia; fatal or non‐fatal myocardial infarction, angina, heart failure, stroke, or kidney failure; death from peripheral vascular disease; amputation; and severe retinopathy (blindness, vitreous haemorrhage or retinal photocoagulation). Events were identified using HES and death registry data (primary cause only), with additional capture of kidney failure from primary care codes.

We assessed incidence rates of heart failure and kidney failure following second‐line therapy initiation separately, given the established benefits of SGLT2i for both endpoints [25, 26]. We also evaluated diabetic ketoacidosis (DKA) as a separate outcome, given the known risk associated with SGLT2i use [27].

Complication outcomes were evaluated for patients initiating second‐line therapy between January 2019 and December 2022 with follow‐up until the earliest of: complication occurrence, discontinuation of second‐line therapy, date of practice deregistration/death, the end of study period (March 2023; end of available follow‐up in HES), or 1 year after treatment initiation.

### 2.3 Statistical Analysis

To evaluate prescribing trends for each drug class, we calculated the proportion of new second‐line initiations for each drug class as a percentage of total second‐line initiations for each calendar year.

#### 2.3.1 Glycaemic, Weight and Treatment Discontinuation Outcomes

We evaluated non‐linear time trends in glycaemic response and weight change for each calendar year using linear regression, with calendar year as a categorical covariate and an interaction between calendar year and age/frailty category. We included all individuals with valid baseline and outcome measures (Flowchart S1) informed by previous work showing limited impact of imputing missing predictor values in this cohort [28]. Estimates were adjusted for a standard covariate set comprising baseline HbA_1c_ (or baseline weight for weight outcome), age at therapy initiation, duration of diabetes, sex, ethnicity and deprivation. Missing ethnicity data were imputed using multiple imputation by chained equations [29]. Treatment discontinuation was evaluated using logistic regression, adjusted for the same covariates.

#### 2.3.2 Severe Diabetes‐Related Complications

Adjusted incidence rates of severe diabetes‐related complications, hospitalisation for heart failure and kidney failure were estimated using Poisson regression, with log follow‐up time (in years) included as an offset term. The model was adjusted for the standard covariate set as well as prior history of the complication (composite, as previously defined). Due to the low number of events, only crude incidence rates were reported for DKA outcome.

To evaluate how clinical outcomes changed for each calendar year, independent of changes in the characteristics of patients initiating second‐line therapy each year, we generated adjusted predictions from the fitted regression models. For each individual, we estimated the counterfactual outcome they would have if they had initiated therapy in each calendar year [30], holding all other covariates constant at their observed values. This produced a set of standardised predictions for each year to estimate expected outcomes, assuming no changes in the underlying population. We summarised overall temporal changes by comparing the adjusted predicted outcomes between the baseline year (2019) and the last year of follow‐up (2022 or 2023, as appropriate). Interpretation of outcome differences between age and frailty groups is limited by differences in baseline complication risk. Our analyses therefore focused on temporal trends within each group, rather than on direct comparisons between groups.

#### 2.3.3 Impact of 2022 NICE Guideline Update

We further evaluated the impact on SGLT2i prescribing following the release of the 2022 UK NICE guidelines, which recommended SGLT2i use independent of glycaemic control for patients with CVD or CKD [4]. Monthly SGLT2i prescribing rates were calculated as a proportion of all second‐line initiations. We used an interrupted time series (ITS) model to estimate pre‐guideline trends, immediate changes following guideline introduction and post‐guideline trends [31]. We tested for autocorrelation using the Durbin–Watson test [32]. A first‐order lag term was included to account for autocorrelation between residuals. Counterfactual predictions were generated to estimate prescribing trends in the absence of the 2022 NICE guidelines.

All analyses used R (v4.4.0) [29, 33, 34].

### 2.4 Sensitivity Analysis

To explore how prescribing trend changes related to patient characteristics beyond age and frailty, we further assessed trends across subgroups defined by sex, ethnicity, deprivation and among patients with or without pre‐existing CVD/CKD (stage 3–4).

To assess severe frailty, we repeated all analyses with the frail > 70 years group stratified by moderate frailty (0.24 < eFI score ≤ 0.36) and severe frailty (eFI score > 0.36).

We repeated glycaemic response and weight change analyses using change from baseline to 6 months (closest ±3 months) and similarly assessed treatment discontinuation within 6 months.

We ran further exploratory analysis of heart failure hospitalisations following second‐line therapy initiation in individuals with pre‐existing heart failure or cardiovascular disease. This aimed to assess whether short‐term population‐level benefits of cardioprotective therapies like SGLT2i were observed in those at highest baseline risk.

## 3 Results

### 3.1 Study Population

Between 2019 and 2024, 117 046 individuals with type 2 diabetes initiated second‐line glucose‐lowering therapy. Of these, 84 589 (72.3%) were aged ≤ 70 years, 18 933 (16.2%) were non‐frail > 70 years and 13 524 (11.5%) were frail > 70 years (Flowchart S1). Table 1 shows the baseline characteristics of the study population by age and frailty status at second‐line treatment initiation. Additional baseline characteristics by calendar year of therapy initiation and by frailty further stratified into moderate and severe frailty are provided in Tables S1 and S2, respectively.

**TABLE 1:** Baseline characteristics of the study cohort at second‐line treatment initiation.

Variable	Age ≤ 70 years	Non‐frail > 70 years	Frail > 70 years
*N* (%)	84 589 (72.3)	18 933 (16.2)	13 524 (11.5)
Age at therapy initiation (years)	55.5 [9.6]	76.2 [4.7]	79.8 [6.0]
Sex (% Male)	50 489 (59.7)	11 625 (61.4)	6967 (51.5)
Duration of diabetes (years)	5.1 [4.3]	8.4 [5.4]	10.2 [5.9]
Ethnicity (%)			
White	64 398 (76.1)	17 063 (90.1)	12 218 (90.3)
South Asian	11 959 (14.1)	851 (4.5)	796 (5.9)
Black	4578 (5.4)	391 (2.1)	272 (2.0)
Mixed	992 (1.2)	103 (0.5)	53 (0.4)
Other	1596 (1.9)	274 (1.4)	89 (0.7)
Missing	1066 (1.3)	251 (1.3)	96 (0.7)
IMD quintile (%)			
1 (least deprived)	9810 (11.6)	3584 (18.9)	2165 (16.0)
2	11 335 (13.4)	3531 (18.6)	2312 (17.1)
3	12 630 (14.9)	3083 (16.3)	2162 (16.0)
4	15 830 (18.7)	2727 (14.4)	2223 (16.4)
5 (most deprived)	18 463 (21.8)	2338 (12.3)	2200 (16.3)
Missing	16 521 (19.5)	3670 (19.4)	2462 (18.2)
Clinical features			
BMI (kg/m^2^)	34.2 [7.7]	30.3 [5.9]	30.6 [6.5]
Weight (kg)	98.5 [23.5]	85.4 [17.8]	83.8 [18.9]
HbA_1c_ (mmol/mol)	74.3 [20.3]	68.7 [18.5]	66.4 [19.9]
Comorbidities (%)			
CVD^a^	15 091 (17.8)	5397 (28.5)	8365 (61.9)
CKD stage 3–4	2709 (3.2)	4191 (22.1)	6254 (46.2)
CKD stage 5	258 (0.3)	49 (0.3)	83 (0.6)
Heart failure	4485 (5.3)	1607 (8.5)	4863 (36.0)
Hyperglycaemia	171 (0.2)	22 (0.1)	30 (0.2)
Hypoglycaemia	52 (0.1)	12 (0.1)	15 (0.1)
Lower limb amputation	179 (0.2)	38 (0.2)	80 (0.6)
Severe retinopathy^b^	192 (0.2)	109 (0.6)	112 (0.8)
eFI category^c^ (%)			
Fit	45 328 (53.6)	5554 (29.3)	0.0
Mild	30 701 (36.3)	13 379 (70.7)	0.0
Moderate	7227 (8.5)	0.0	8961 (66.3)
Severe	1333 (1.6)	0.0	4563 (33.7)

*Note:* Values for continuous variables are given as mean [SD] and binary variables as *n* (%).

^a^ CVD: myocardial infarction, stroke, revascularisation, ischaemic heart disease, angina, peripheral arterial disease, transient ischaemic attack.

^b^ Severe retinopathy: vitreous haemorrhage, retinal photocoagulation.

^c^ eFI category: fit (eFI ≤ 0.12); mild frailty (0.12 < eFI ≤ 0.24); moderate frailty (0.24 < eFI ≤ 0.36); severe frailty (eFI > 0.36).

### 3.2 Major Increase in SGLT2i Prescriptions Across Age and Frailty Subgroups Over 2019–2024

**FIGURE 1:**
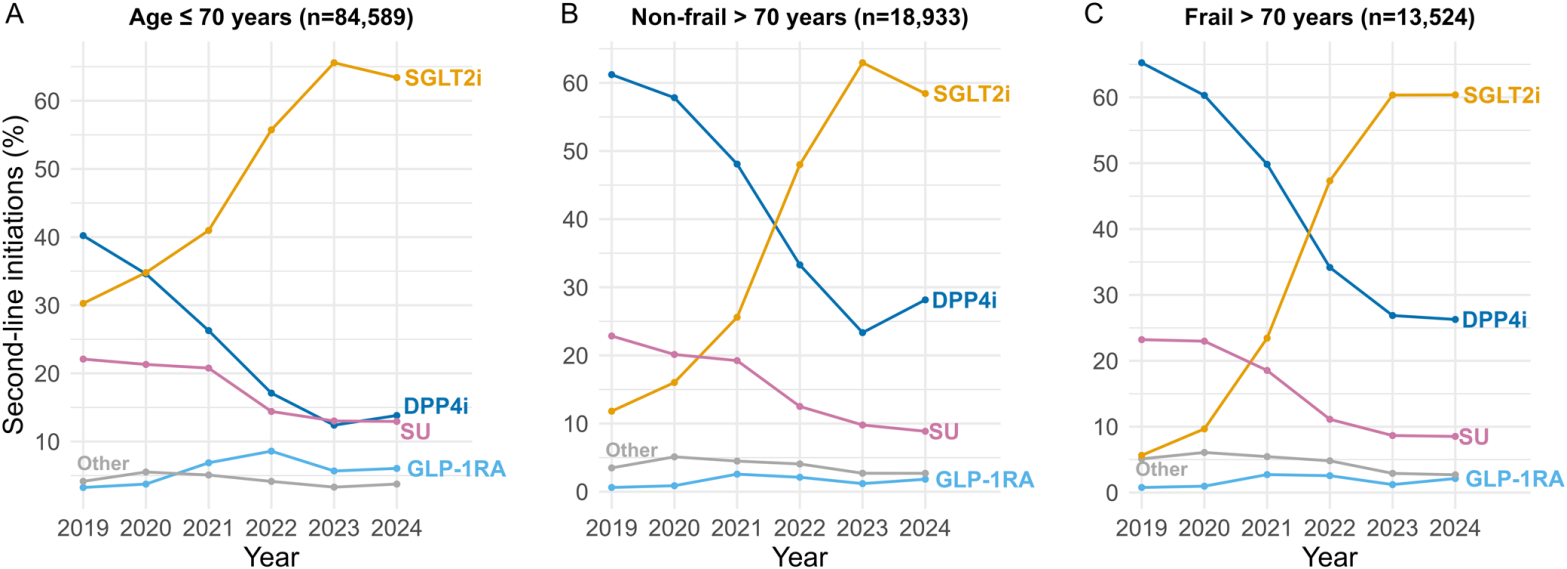
Time trends from 2019 to 2024* in new second‐line drug therapy after metformin in patients (A) aged ≤ 70 years (*n* = 84 589), (B) non‐frail > 70 years (*n* = 18 933) and (C) frail > 70 years (*n* = 13 524). The prescriptions for each drug class each year are given as the percentage of total new drug prescriptions for second‐line therapies that year. *2024 data include January to March.

We observed substantial changes in second‐line prescribing between 2019 and 2024, with SGLT2i becoming the most initiated second‐line therapy across all age and frailty subgroups. Overall, the proportion of SGLT2i prescriptions increased markedly from 26% of initiations in 2019 to 63% in 2024. This increase was most marked in those frail > 70 years where the proportion of SGLT2i prescriptions increased 10‐fold from 6% in 2019 to 60% in 2024 (Figure 1C). Similar trends were observed in those non‐frail > 70 years (12% in 2019 vs. 58% in 2024; Figure 1B) and in those aged ≤ 70 (30% in 2019 vs. 63% in 2024; Figure 1A).

During the study period, SGLT2i overtook DPP4i and sulfonylureas, becoming the most common second‐line therapy across all age and frailty groups, with DPP4i use declining overall from 44% of second‐line initiations in 2019 to 16% in 2024 and SU use from 22% to 13%. We observed no increase in second‐line GLP‐1RA initiations from 2019 to 2024.

Prescribing trends were similar across subgroups defined by sex, ethnicity, deprivation, in patients with and without pre‐existing CVD or CKD and across frailty subgroups further stratified by moderate and severe frailty, with SGLT2i as the most initiated therapy for all subgroups in 2024 (Figures S1–S6).

### 3.3 Changes in Prescribing Were Temporally Associated With Modest Improvements in HbA_1c_ and Weight Response Across Age and Frailty Subgroups, Without Change in Treatment Discontinuation

**FIGURE 2:**
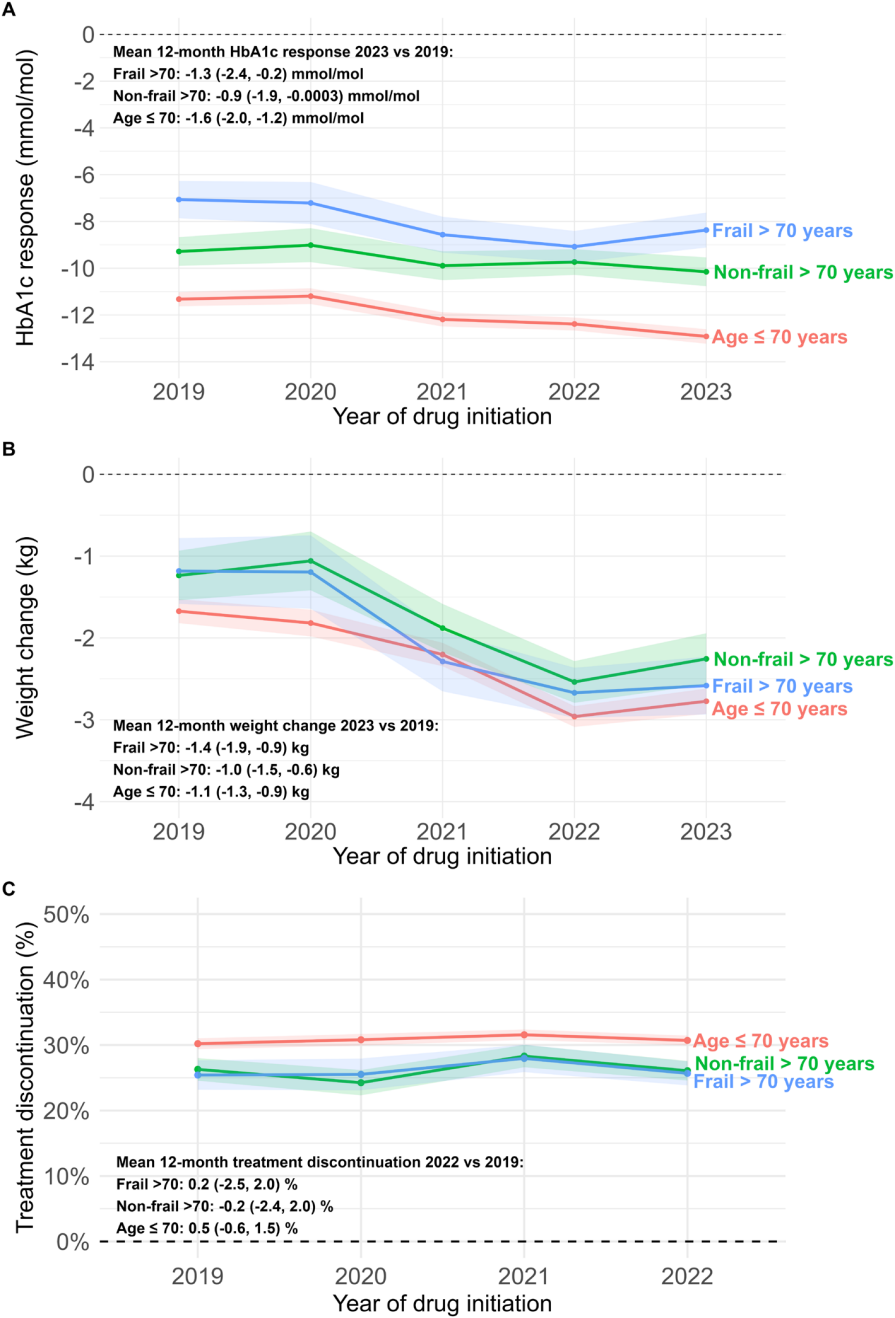
Adjusted mean 12‐month response following second‐line therapy initiation for (A) HbA1c (mmol/mol) and (B) weight (kg) 2019 to 2023 and (C) treatment discontinuation within 12‐months of second‐line therapy initiation 2019–2022, by age and frailty subgroups. Analyses included individuals with valid baseline and eligible 12‐month follow‐up measurements for HbA1c (*n* = 53 737 [46%] of *n* = 117 046) and weight (*n* = 50 535 [43%] of *n* = 117 046). Discontinuation analyses included individuals who either remained on second‐line therapy for ≥ 12 months or discontinued and had at least ≥ 3 months' follow‐up after their final prescription to confirm the drug was discontinued (*n* = 79 296 [68%] of *n* = 117 046 included). Shading represents 95% confidence intervals.

Over 2019–2023, we found modest improvements in glycaemic response and weight change following second‐line therapy initiation across all age and frailty subgroups (Figure 2A,B). In patients aged ≤ 70 years, mean 12‐month HbA_1c_ response following initiation of second‐line therapy improved from −11.3 mmol/mol (95% CI −11.6 to −11.0) in 2019 to −12.9 mmol/mol (95% CI −13.2 to −12.6) in 2023, an improvement of −1.6 mmol/mol (95% CI −2.0 to −1.2; *p* < 0.001 for 2023 vs. 2019 difference; Figure 2A). Similar trends were also seen in frail and non‐frail patients > 70 years, although average response was greater in patients aged ≤ 70 years.

Weight reductions over the same period followed a similar trend (Figure 2B). In patients aged ≤ 70 years, mean 12‐month weight change following initiation of second‐line therapy improved from −1.7 kg (95% CI −1.8 to −1.5) in 2019 to −2.8 kg (95% CI −2.9 to −2.6) in 2023, a difference of −1.1 kg (95% CI −1.3 to −0.9; *p* < 0.001 for 2023 vs. 2019 difference). Similar trends were observed in frail and non‐frail patients > 70 years.

The risk of treatment discontinuation following initiation of second‐line therapy remained stable (Figure 2C). In patients aged ≤ 70 years, the risk of discontinuation following second‐line therapy initiation was 30.2% (95% CI 29.4 to 31.0) in 2019 compared to 30.7% in 2022 (95% CI 30.0 to 31.4), a difference in risk of 0.5% (95% CI −0.6 to 1.6; *p* = 0.36 for 2022 vs. 2019 difference). We observed no evidence of change in treatment discontinuation for frail and non‐frail patients > 70 years.

Time trends for glycaemic response, weight change and treatment discontinuation were consistent in sensitivity analyses using a 6‐month instead of a 12‐month window (Figure S7) and when further stratifying frailty status by moderate and severe frailty (Figure S8).

### 3.4 Limited Evidence of Changes Over Time in Incidence of Short‐Term Severe Diabetes Complications, Heart Failure, Kidney Failure, or DKA

**FIGURE 3:**
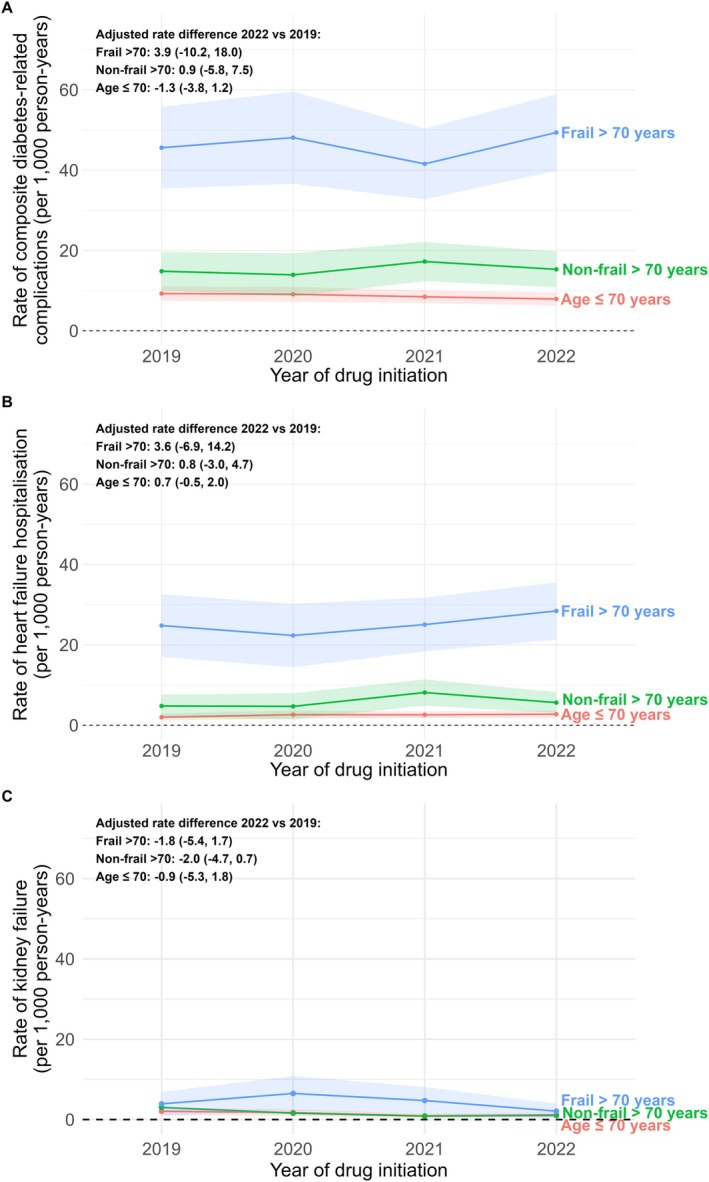
Adjusted incidence rates of (A) a severe diabetes‐related complication (sudden death; death due to hyperglycaemia or hypoglycaemia; fatal or non‐fatal myocardial infarction, angina, heart failure, stroke, or kidney failure; death from peripheral vascular disease; amputation; and severe retinopathy [blindness, vitreous haemorrhage or retinal photocoagulation]), (B) heart failure and (C) kidney failure following initiation of second‐line therapy, stratified by age and frailty subgroups. Analyses for all complication outcomes included *n* = 63 965 individuals with linked HES data. Shaded areas represent 95% confidence intervals. Underlying crude incidence rates for all complication outcomes are reported in Table S3.

In adults aged ≤ 70 years, the adjusted incidence rate of severe diabetes‐related complications was stable over 2019–2022, 2019: 9.9 (95% CI 8.1 to 11.7; *p* < 0.001); 2022: 8.6 (95% CI 6.9 to 10.3) per 1000 person‐years; difference: −1.3 (95% CI −3.8 to 1.2; *p* = 0.31; Figure 3A). Heart failure incidence was similarly stable, 2019: 2.0 (95% CI 1.1 to 2.9), 2022: 2.7 (95% CI 1.8 to 3.7); difference: 0.7 (95% CI −0.5 to 2.0; *p* = 0.20; Figure 3B), including in those with pre‐existing heart failure or cardiovascular disease (Figure S9). Kidney failure incidence was also stable, 2019: 2.1 (95% CI 1.1 to 3.0); 2022: 1.2 (95% CI 0.5 to 1.8); difference −0.9 (95% CI −2.0 to 0.3; *p* = 0.13; Figure 3C). Although adjusted incidence rates were higher for all outcomes, stable trends by calendar year were consistently observed in frail and non‐frail patients > 70 years.

Incidence of DKA was rare (< 50 events total across subgroups from 2019 to 2022) with no evidence of meaningful change by calendar year (Table S3).

### 3.5 Impact of UK NICE 2022 Guideline Changes on SGLT2i Initiation

**FIGURE 4:**
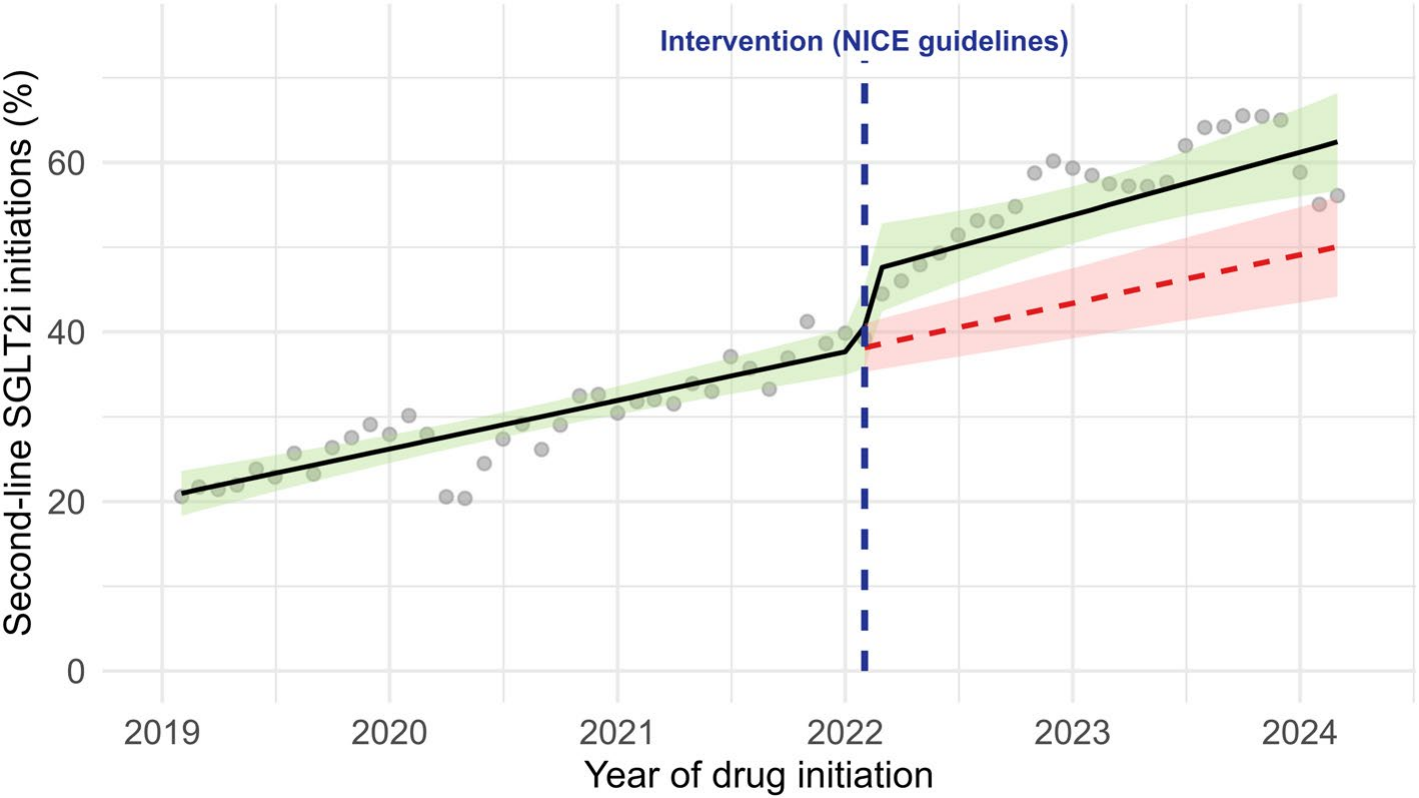
Interrupted time series analysis of monthly SGLT2‐inhibitor initiations, 2019–2024. The solid black line shows the observed trend, the dashed red line shows the counterfactual trend and the vertical dashed blue line shows the intervention (publication of the NICE guidelines). Shading represents 95% confidence intervals.

Overall, monthly SGLT2i initiations were already increasing before the 2022 NICE guidelines by 0.55% per month (95% CI 0.35 to 0.74; *p* < 0.001) (Figure 4). In the month following publication, there was an immediate 7.5% increase in monthly initiations (95% CI 3.1 to 11.9; *p* < 0.001). We observed similar trends in both frail and non‐frail adults > 70 years (Figure S10).

## 4 Discussion

This population‐based cohort study reveals a major shift in early initiation of glucose‐lowering therapies in the UK between 2019 and 2024, with SGLT2i becoming the most common second‐line therapy even in frail older adults. SGLT2i have largely replaced DPP4i and sulfonylureas. Over the same time period we observed modest population‐level improvements in HbA_1c_ response and weight loss after treatment initiation, but no evidence of changes in short‐term treatment discontinuation or rates of severe diabetes‐related complications, heart failure, kidney failure, or DKA within 12 months of treatment initiation. Importantly, these trends in short‐term outcomes were consistent in frail older adults during a period in which the proportion of SGLT2i initiations increased 10‐fold in this group.

The increased early prescribing of SGLT2i represents a clear change from earlier UK data, where DPP4i were the most commonly initiated second‐line therapy overall and in people with cardiorenal comorbidity, despite an absence of cardiorenal benefit and comparable cost to SGLT2i [6, 16, 17]. New 2026 UK NICE guidance now recommends SGLT2i alongside modified‐release metformin as first‐line therapy for all people with type 2 diabetes except those with frailty or adverse events [12]. Our results provide meaningful real‐world evidence to complement this recommendation by showing that early use of SGLT2i has already increased markedly even in those with frailty in the UK, without evidence of corresponding population‐level increases in short‐term discontinuation or severe diabetes complications. Importantly, these results were consistent even in those with severe frailty. Our finding of an immediate increase in SGLT2i initiations following the previous 2022 NICE guidance update suggests that the recent changes in recommendations may rapidly influence UK clinical practice.

Beyond the UK, recent studies from Europe and North America have also reported increasing use of SGLT2i and GLP‐1RA [15, 35, 36, 37], although generally not to the extent observed in our UK cohort (10‐fold increase in SGLT2i initiation among frail older adults). Analysis from other countries, including Italy and Canada [35, 36], has shown continued lower uptake of both drug classes in older adults. These comparisons suggest that the UK may be further ahead in adopting SGLT2i as second‐line therapy, including in older adults with frailty and that our findings may help inform implementation in other healthcare systems.

In contrast to the marked change in SGLT2i initiations, we did not observe an increase in second‐line GLP‐1RA use, despite trial evidence of cardiovascular and renal benefit [38]. This likely reflects UK guidance, where historically GLP‐1RA have been primarily recommended as third‐line therapies and limited to individuals with a BMI ≥ 35 kg/m^2^ [4]. The latest UK guidance now recommends GLP‐1RA much earlier in the type 2 diabetes treatment pathway, including as potential first‐line therapy alongside metformin and SGLT2i for those with young‐onset diabetes diagnosed under age 40 and those with existing cardiovascular disease [12]. Future work should assess the real‐world impact of these changes, including identification of prescribing disparities and risk/benefit across population subgroups beyond those included in clinical trials.

In supplementary analyses, early SGLT2i initiation was broadly similar across sociodemographic and clinical subgroups, including ethnicity, deprivation, CVD and CKD. This suggests reported UK disparities in SGLT2i use may reflect differences in treatment intensification rather than prescribing choice at intensification [39]. Further studies are needed to confirm this and guide interventions to reduce inequalities [40].

Although we did not evaluate drug‐specific effects in this study, modest population‐level improvements in HbA_1c_ and weight are consistent with the replacement of DPP4i prescribing for SGLT2i. Clinical trials have consistently shown greater glycaemic and weight benefits with SGLT2i compared with DPP4i and the effects observed in our study (mean HbA_1c_ reduction of −1.6 mmol/mol and mean weight loss of −1.1 kg) align with these findings [41, 42, 43, 44, 45, 46]. Although modest at the individual level, a −1.6 mmol/mol greater average HbA_1c_ reduction has meaningful population‐level implications. Given that the average rate of glycaemic progression in type 2 diabetes is approximately 1 mmol/mol per year, a 1.6 mmol/mol benefit translates to an average 1.6 additional years of stable glycaemic control before treatment intensification may be required [47]. In the future, implementation of precision medicine approaches, such as a recently developed model to predict optimal glucose‐lowering therapy considering five major drug classes after metformin [48], could provide an alternative to current prescribing practice by allowing targeting of therapy to optimise glycaemic response for an individual patient based on their routine clinical features.

During a period of marked increase in SGLT2i prescribing, we did not find evidence of meaningful population‐level changes in rates of short‐term complications including heart failure and end‐stage kidney failure, despite the known benefits of SGLT2i for these outcomes [49]. Several factors may explain this. First, as our focus was on evaluating temporal trends, complication outcomes were limited by a 12‐month follow‐up window following second‐line treatment initiation. Although cardiovascular and renal benefits of SGLT2i have been observed in trials within the first year [50], participants have been predominantly those with pre‐existing cardiovascular and kidney disease in contrast to our lower risk population‐based cohort. Supporting this, a recent meta‐analysis found the absolute risk reduction with SGLT2i for hospitalisation for heart failure was substantially greater in those with established cardiovascular disease than those without [51]. The unavailability of hospital admission data beyond 2023 also meant we lacked data on complication outcomes during the period of highest SGLT2i uptake. Second, because our outcome analyses included all second‐line therapies, not just SGLT2i, even in the most recent years any overall treatment effects may have been attenuated by the 40% of patients who initiated alternative drug classes without cardiovascular and renal benefits. Third, the study period overlapped with the COVID‐19 pandemic which likely affected hospital admissions. Our analysis therefore highlights the need for focused studies evaluating longer‐term real‐world outcomes with SGLT2i as their use across the wider population with type 2 diabetes increases.

DKA is a recognised adverse event with SGLT2i, prompting safety warnings from the European Medicines Agency [52]. In our study, we found no increase in DKA incidence following initiation of second‐line therapy, with events remaining rare even in frail older adults. However, recent UK causal analysis suggests that although absolute rates remain low, the relative risk of DKA is higher with SGLT2i in adults aged ≥ 70 years compared with DPP4i, but not in those < 70 years [27], highlighting the importance of careful prescribing and monitoring in older adults.

Our study has several strengths. We used large, population‐based data from CPRD, which is broadly representative of the UK population by age, sex and ethnicity [53]. A strength of this UK setting is that prescribing occurs within a universal healthcare system, meaning initiation of treatment is unlikely to be directly affected by a patient's ability to pay [23]. We applied a validated electronic frailty index, allowing robust subgroup analyses in frail populations who are often excluded from clinical trials [21]. A limitation of this approach is that individuals aged ≤ 70 years were analysed as a single heterogeneous group. However, this group represents the majority of people initiating second‐line therapy and additional subgroup analyses by sex, ethnicity and deprivation showed broadly consistent prescribing trends. As an observational study, our results simply describe time trends and do not attempt to provide causal evidence that changes in prescribing patterns have led to improved treatment efficacy. Alternative explanations include changes over time in follow‐up intensity, testing frequency, or selection of individuals suitable for treatment escalation. In addition, our adjusted analysis assumes that differences across calendar years reflect temporal effects alone, but changes in patient characteristics, drug adherence, unmeasured confounders including changes in healthcare delivery post‐COVID and missing outcome data may not have been fully accounted for.

In summary, from 2019 to 2024, there has been a substantial shift in second‐line therapy for type 2 diabetes in the UK, with SGLT2i becoming the most commonly initiated treatment and the greatest increase in SGLT2i use in older adults with frailty. Over the same period, short‐term outcomes remained stable, with modest benefits for HbA_1c_ and weight and no meaningful change in treatment discontinuation or major complications including heart failure, kidney failure, or DKA. These findings support the careful use of SGLT2i across the wider population with type 2 diabetes including frail older adults.

## Author Contributions

M.M.D., J.M.D., B.M.S., T.J.M. and K.G.Y. conceived and developed the study concept and design. M.M.D., J.M.D., K.G.Y., P.C., L.M.G., T.T.J. and B.M.S. had access to all the raw datasets used for the study. A.P.M. reviewed the clinical codes. M.M.D. undertook the analysis, with support from K.G.Y., T.J.M., B.M.S. and J.M.D. All authors provided support for the analysis and interpretation of results, critically revised the manuscript and approved the final manuscript. M.M.D. and J.M.D. attest that all listed authors meet authorship criteria and that no others meeting the criteria have been omitted. All authors had final responsibility for the decision to submit for publication.

## Funding

This work was supported by the Medical Research Council (MR/W003988/1), the Wellcome Trust (227070/Z/23/Z) and the NIHR Exeter Biomedical Research Centre. The funders had no role in considering the study design or in the collection, analysis, interpretation of data, writing of the report, or decision to submit the article for publication. This study has been delivered through the National Institute for Health and Care Research (NIHR) Exeter Biomedical Research Centre (BRC). The views expressed are those of the author(s) and not necessarily those of the Medical Research Council, the NIHR or the Department of Health and Social Care. For the purpose of open access, the authors have applied a Creative Commons Attribution (CC BY) licence to any Author Accepted Manuscript version arising from this submission.

## Disclosure

The senior author (J.M.D.) is the guarantor of this manuscript and affirms that the manuscript is an honest, accurate and transparent account of the study being reported; that no important aspects of the study have been omitted; and that any discrepancies from the study as originally planned have been explained. The study protocol was not pre‐registered.

## Ethics Statement

This study was approved by the CPRD independent scientific advisory committee (eRAP 24_004747). CPRD also has ethical approval from the Health Research Authority to support research using anonymised patient data (research ethics committee reference 21/EM/0265). Individual patient consent was not required as all data were deidentified.

## Conflicts of Interest

J.M.D. is supported by a Wellcome Trust Early Career award (227 070/Z/23/Z). A.T.H. and B.M.S. are supported by the NIHR Exeter Clinical Research Facility; the views expressed are those of the authors and not necessarily those of the NHS, the NIHR or the Department of Health. A.P.M. declares previous research funding from Eli Lilly, Pfizer and AstraZeneca. A.G.J. declares research funding to his university from the UK Medical Research Council, NIHR, Diabetes UK, Breakthrough Type 1 diabetes, the Novo Nordisk Foundation and European Foundation for the Study of Diabetes. E.R.P. has received honoraria for speaking from Lilly, Novo Nordisk and Illumina. No industry representatives were involved in the writing of the manuscript or analysis of data. For all authors these are outside the submitted work; there are no other relationships or activities that might bias, or be perceived to bias, their work.

## Supporting information


**Table S1:** Baseline characteristics of the study cohort at second‐line treatment initiation by calendar year (2019–2024).
**Table S2:** Baseline characteristics of the study cohort at second‐line treatment initiation with age and frailty subgroups further stratified into moderate and severe frailty.
**Table S3:** Crude incidence rates of complications following second‐line therapy initiation including a severe diabetes‐related complication, heart failure, kidney failure and DKA, per 1000 person‐years by calendar year (2019–2022) and frailty category a) age ≤ 70 (*n* = 84 589) b) non‐frail > 70 (*n* = 18 933) c) frail > 70 (*n* = 13 524).
**Flowchart S1**. CPRD patient flow and inclusion criteria for individuals initiating second‐line glucose‐lowering therapy and those included in each analysis.
**Figure S1:** Trends in second‐line initiations by sex (2019–2024).
**Figure S2:** Trends in second‐line initiations by ethnicity (2019–2024).
**Figure S3:**. Trends in second‐line initiations by deprivation (IMD quintiles) (2019–2024).
**Figure S4:** Trends in second‐line initiations by baseline cardiovascular disease status (2019–2024).
**Figure S5:** Trends in second‐line initiations by baseline chronic kidney disease status (2019–2024).
**Figure S6:** Trends in second‐line initiations by age and frailty subgroup (2019–2024), with frailty further categorised into moderate and severe.
**Figure S7:** 6‐month HbA1c response, weight change (2019–2023) and treatment discontinuation (2019–2022) following second‐line therapy initiation by age and frailty subgroups.
**Figure S8:** 12‐month HbA1c response, weight change (2019–2023) and treatment discontinuation (2019–2022) following second‐line therapy initiation by age and frailty subgroups, with frailty further categorised into moderate and severe.
**Figure S9:** Rate (per 1000 person‐years) following second‐line therapy initiation of heart failure hospitalisations in patients with pre‐existing heart failure or cardiovascular disease across age and frailty subgroups.
**Figure S10:** Interrupted time series analysis of monthly SGLT2i initiations following 2022 NICE guideline update by age and frailty subgroups, 2019–2024. The solid black line shows the observed trend, the dashed red line shows the counterfactual trend and the vertical dashed blue line shows the intervention (publication of the NICE guidelines).

## Data Availability

Access to CPRD data is subject to protocol approval via CPRD's research data governance process (https://cprd.com/data‐access). Code for initial cohort preparation is available at: https://github.com/Exeter‐Diabetes/CPRD‐Cohort‐scripts/tree/main/03‐Treatment‐response‐(MASTERMIND) and analysis code is available at: https://github.com/Exeter‐Diabetes/CPRD‐Martha‐prescribing‐trends.
