# Supplementary material for: Trends in Second‐Line Initiations and Treatment Outcomes Across Age and Frailty Groups in People With Type 2 Diabetes: UK Population‐Based Study, 2019–2024
Source: Diabetes Obes Metab. 2026 Jun 4;28(8):7471–82. doi: 10.1111/dom.70960 (PMC13341369; doi:10.1111/dom.70960)

Supplementary material for:

Trends in second-line initiations and treatment outcomes across age and frailty groups in people with type 2 diabetes: UK population-based study, 2019-2024

Authors: MM Dinsdale, KG Young, P Cardoso, LM Güdemann, TT Jansz, AP McGovern, AG Jones, ER Pearson, AT Hattersley, TJ McKinley, BM Shields, JM Dennis

Supplementary Flowchart. CPRD patient flow and inclusion criteria for individuals initiating second-line glucose-lowering therapy and those included in each analysis

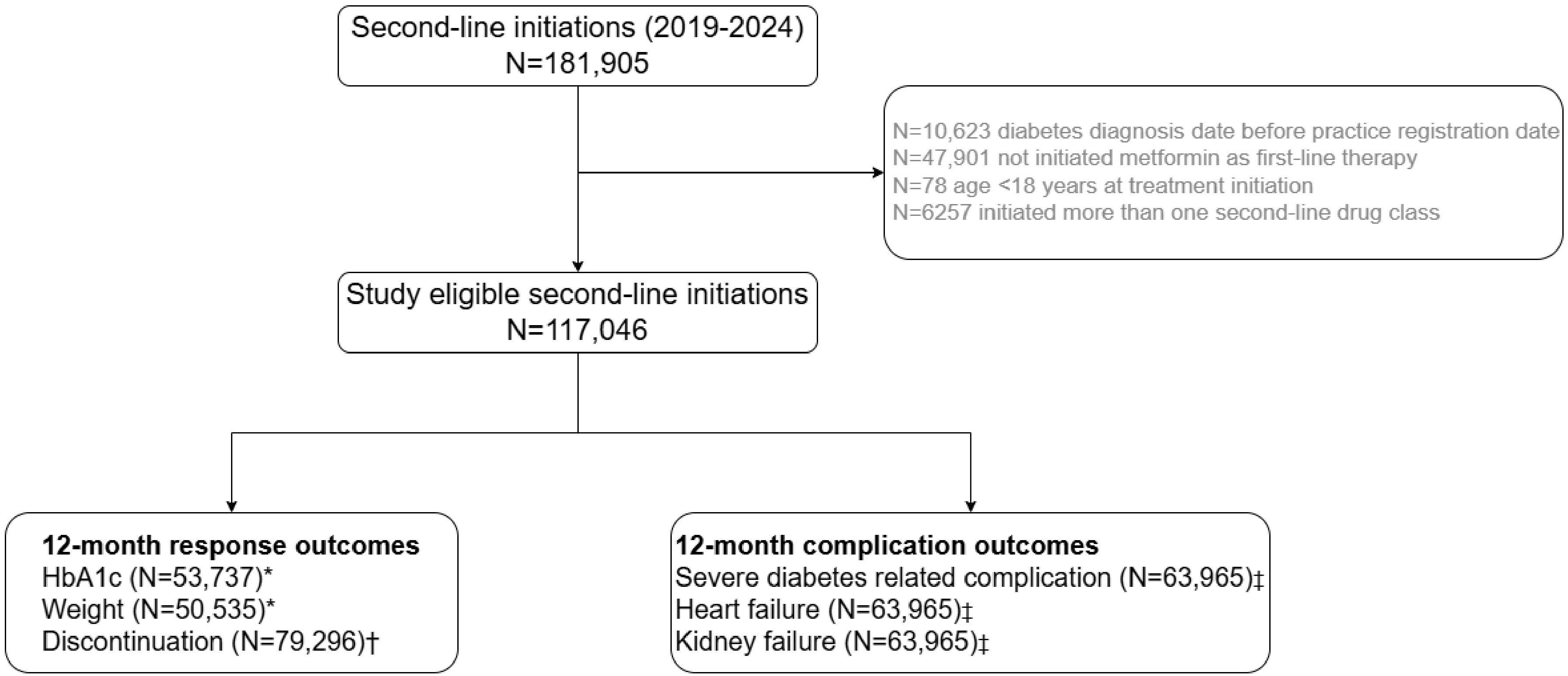

*N [%] with valid data for 12-month analysis. Each outcome was defined as the closest recorded value to 12 months post-treatment initiation +/- 3 months

†For treatment discontinuation we included all individuals who stopped second-line therapy within 12 months of initiation with ≥3 months follow-up time available after their last prescription (to confirm that the drug of interest was discontinued).

‡ For complication analyses we included all individuals with linked HES data and valid follow-up time after initiating second-line therapy

Supplementary Table 1. Baseline characteristics of the study cohort at second-line treatment initiation by calendar year (2019-2024).

	**2019**	**2020**	**2021**	**2022**	**2023**	**2024**
Second-line initiations	19354	16391	20918	24632	29569	6182
Age at therapy initiation (years)	61.4 [13.1]	60.7 [13.2]	61.5 [13.2]	62.0 [13.2]	62.1 [13.2]	62.1 [13.2]
Sex (% Male)	11356 (58.7)	9615 (58.7)	12163 (58.1)	14,549 (59.1)	17,635 (59.6)	3763 (60.9)
Duration of diabetes (years)	6.0 [4.1]	5.9 [4.8]	6.2 [5.0]	6.4 [5.0]	6.4 [5.3]	6.5 [5.4]
**Ethnicity (%)**						
White	15520 (80.2)	13085 (79.8)	16873 (80.7)	19881 (80.7)	23480 (79.4)	4840 (78.3)
South Asian	2338 (12.1)	1886 (11.5)	2296 (11.0)	2727 (11.1)	3577 (12.1)	782 (12.6)
Black	759 (3.9)	763 (4.7)	992 (4.7)	1076 (4.4)	1338 (4.5)	313 (5.1)
Mixed	189 (1.0)	179 (1.1)	208 (1.0)	219 (0.9)	299 (1.0)	54 (0.9)
Other	278 (1.4)	257 (1.6)	307 (1.5)	497 (2.0)	516 (1.7)	104 (1.7)
Missing	270 (1.4)	221 (1.3)	242 (1.2)	232 (0.9)	359 (1.2)	89 (1.4)
**IMD quintile (%)**						
1 (least deprived)	2565 (13.3)	2074 (12.7)	2848 (13.6)	3330 (13.5)	3916 (13.2)	826 (13.4)
2	2924 (15.1)	2436 (14.9)	3037 (14.5)	3644 (14.8)	4243 (14.3)	893 (14.4)
3	3002 (15.5)	2425 (14.8)	3149 (15.1)	3808 (15.5)	4539 (15.4)	952 (15.4)
4	3465 (17.9)	3014 (18.4)	3805 (18.2)	4358 (17.7)	5113 (17.3)	1026 (16.6)
5 (most deprived)	3828 (19.8)	3352 (20.5)	4269 (20.4)	4808 (19.5)	5589 (18.9)	1155 (18.7)
Missing	3570 (18.4)	3090 (18.9)	3810 (18.2)	4684 (19.0)	6169 (20.9)	1330 (21.5)
**Clinical features**						
BMI (kg/m^2)	32.8 [7.2]	33.1 [7.3]	33.3 [7.5]	33.3 [7.6]	33.1 [7.5]	33.0 [7.4]
Weight (kg)	93.6 [22.5]	94.9 [22.7]	95.2 [23.1]	95.0 [23.6]	94.7 [23.1]	94.5 [23.2]
HbA1c (mmol/mol)	72.3 [19.1]	76.0 [20.8]	75.2 [20.7]	72.1 [19.9]	69.7 [19.8]	70.0 [20.0]
**Comorbidities (%)**						
CVD[a]	4531 (23.4)	3901 (23.8)	5271 (25.2)	6295 (25.6)	7368 (24.9)	1487 (24.1)
CKD stage 3-4	2226 (11.5)	1788 (10.9)	2400 (11.5)	2898 (11.8)	3222 (10.9)	620 (10.0)
CKD stage 5	70 (0.4)	56 (0.3)	70 (0.3)	85 (0.3)	92 (0.3)	17 (0.3)
Heart failure	1410 (7.3)	1306 (8.0)	2044 (9.8)	2760 (11.2)	2951 (10.0)	566 (9.2)
Hyperglycaemia	37 (0.2)	52 (0.3)	53 (0.3)	55 (0.2)	25 (0.1)	2 (0.0)
Hypoglycaemia	13 (0.1)	13 (0.1)	20 (0.1)	21 (0.1)	10 (0.0)	2 (0.0)
Lower limb amputation	42 (0.2)	46 (0.3)	73 (0.3)	61 (0.2)	65 (0.2)	13 (0.2)
Severe retinopathy[b]	57 (0.3)	69 (0.4)	70 (0.3)	94 (0.4)	107 (0.4)	16 (0.3)
**Drug class (%)**						
DPP4i	8975 (46.4)	6678 (40.7)	6786 (32.4)	5384 (21.9)	4727 (16.0)	1091 (17.6)
SGLT2i	4756 (24.6)	4814 (29.4)	7640 (36.5)	13,162 (53.4)	19,074 (64.5)	3847 (62.2)
GLP-1RA	495 (2.6)	498 (3.0)	1197 (5.7)	1672 (6.8)	1299 (4.4)	303 (4.9)
SU	4324 (22.3)	3495 (21.3)	4242 (20.3)	3375 (13.7)	3534 (12.0)	727 (11.8)
Other	804 (4.2)	906 (5.5)	1053 (5.0)	1039 (4.2)	935 (3.2)	214 (3.5)

Values for continuous variables are given as mean [SD] and binary variables as n (%)

[a]CVD: myocardial infarction, stroke, revascularisation, ischaemic heart disease, angina, peripheral arterial disease, transient ischaemic attack. [b]Severe retinopathy: vitreous haemorrhage, retinal photocoagulation

Supplementary Table 2. Baseline characteristics of the study cohort at second-line treatment initiation with age and frailty subgroups further stratified into moderate and severe frailty.

Variable	Age ≤ 70 years	Non-frail >70 years	Moderate >70 years	Severe >70 years
N (%)	84589 (72.3)	18933 (16.2)	8961 (7.6)	4563 (3.9)
Age at therapy initiation (years)	55.5 [9.6]	76.2 [4.7]	78.9 [5.7]	81.5 [6.2]
Sex (% Male)	50489 (59.7)	11625 (61.4)	4889 (54.6)	2018 (44.2)
Duration of diabetes (years)	5.1 [4.3]	8.4 [5.4]	9.8 [5.8]	11.0 [6.0]
**Ethnicity (%)**				
White	64398 (76.1)	17063 (90.1)	8073 (90.1)	4145 (90.8)
South Asian	11959 (14.1)	851 (4.5)	528 (5.9)	268 (5.9)
Black	4578 (5.4)	391 (2.1)	185 (2.1)	87 (1.9)
Mixed	992 (1.2)	103 (0.5)	45 (0.5)	8 (0.2)
Other	1596 (1.9)	274 (1.4)	66 (0.7)	23 (0.5)
Missing	1066 (1.3)	251 (1.3)	64 (0.7)	32 (0.7)
**IMD quintile (%)**				
1 (least deprived)	9810 (11.6)	3584 (18.9)	1498 (16.7)	667 (14.6)
2	11335 (13.4)	3531 (18.6)	1593 (17.8)	719 (15.8)
3	12630 (14.9)	3083 (16.3)	1459 (16.3)	703 (15.4)
4	15830 (18.7)	2727 (14.4)	1458 (16.3)	765 (16.8)
5 (most deprived)	18463 (21.8)	2338 (12.3)	1352 (15.1)	848 (18.6)
Missing	16521 (19.5)	3670 (19.4)	1601 (17.9)	861 (18.9)
**Clinical features**				
BMI (kg/m^2)	34.2 [7.7]	30.3 [5.9]	30.6 [6.3]	30.5 [6.6]
Weight (kg)	98.5 [23.5]	85.4 [17.8]	84.6 [18.8]	82.0 [19.0]
HbA1c (mmol/mol)	74.3 [20.3]	68.7 [18.5]	67.0 [19.6]	65.3 [20.2]
**Comorbidities (%)**				
CVD[a]	15091 (17.8)	5397 (28.5)	5006 (55.9)	3359 (73.6)
CKD stage 3-4	2709 (3.2)	4191 (22.1)	3638 (40.6)	2616 (57.3)
CKD stage 5	258 (0.3)	49 (0.3)	52 (0.6)	31 (0.7)
Heart failure	4485 (5.3)	1607 (8.5)	2518 (28.1)	2345 (51.4)
Hyperglycaemia	171 (0.2)	22 (0.1)	18 (0.2)	12 (0.3)
Hypoglycaemia	52 (0.1)	12 (0.1)	10 (0.1)	5 (0.1)
Lower limb amputation	179 (0.2)	38 (0.2)	33 (0.4)	47 (1.0)
Severe retinopathy[b]	192 (0.2)	109 (0.6)	69 (0.8)	43 (0.9)
**eFI category (%)**				
Fit	45328 (53.6)	5554 (29.3)		
Mild	30701 (36.3)	13379 (70.7)		
Moderate	7227 (8.5)		8961 (100)	
Severe	1333 (1.6)			4563 (100)

Values for continuous variables are given as mean [SD] and binary variables as n (%)

[a]CVD: myocardial infarction, stroke, revascularisation, ischaemic heart disease, angina, peripheral arterial disease, transient ischaemic attack

[b]Severe retinopathy: vitreous haemorrhage, retinal photocoagulation

Supplementary Figure 1. Trends in second-line initiations by sex (2019-2024)

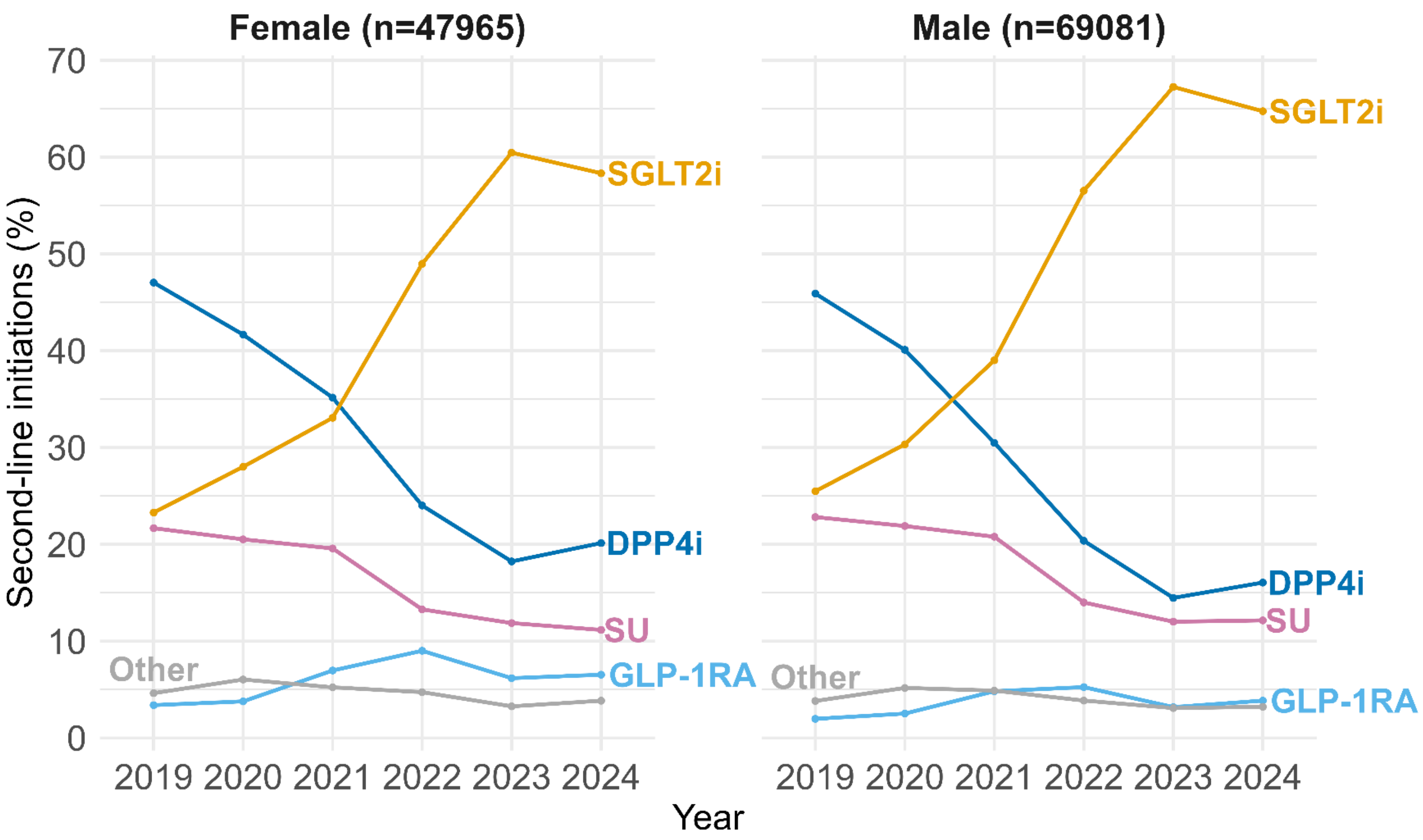

Supplementary Figure 2. Trends in second-line initiations by ethnicity (2019-2024)

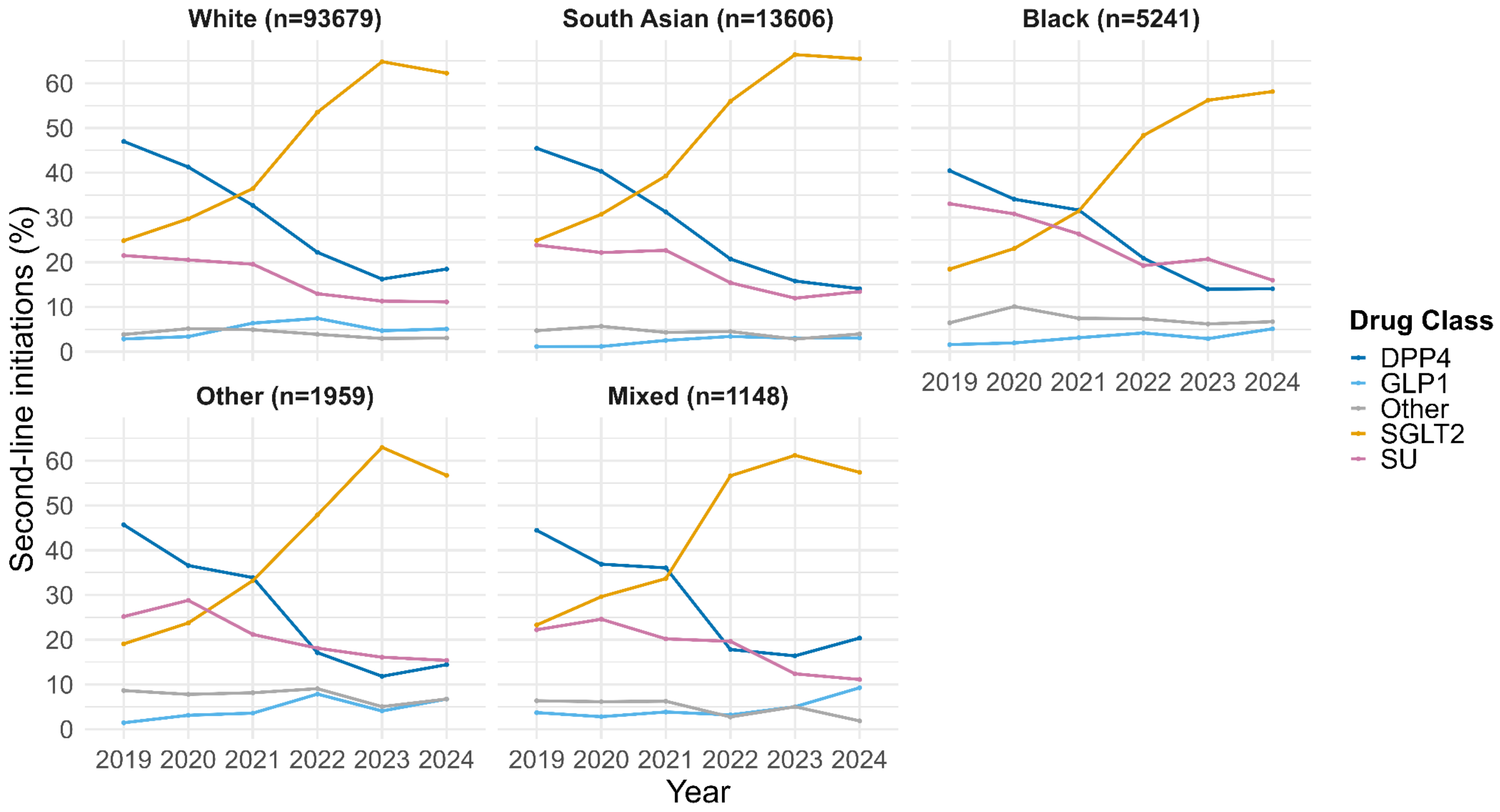

Supplementary Figure 3. Trends in second-line initiations by deprivation (IMD quintiles) (2019–2024)

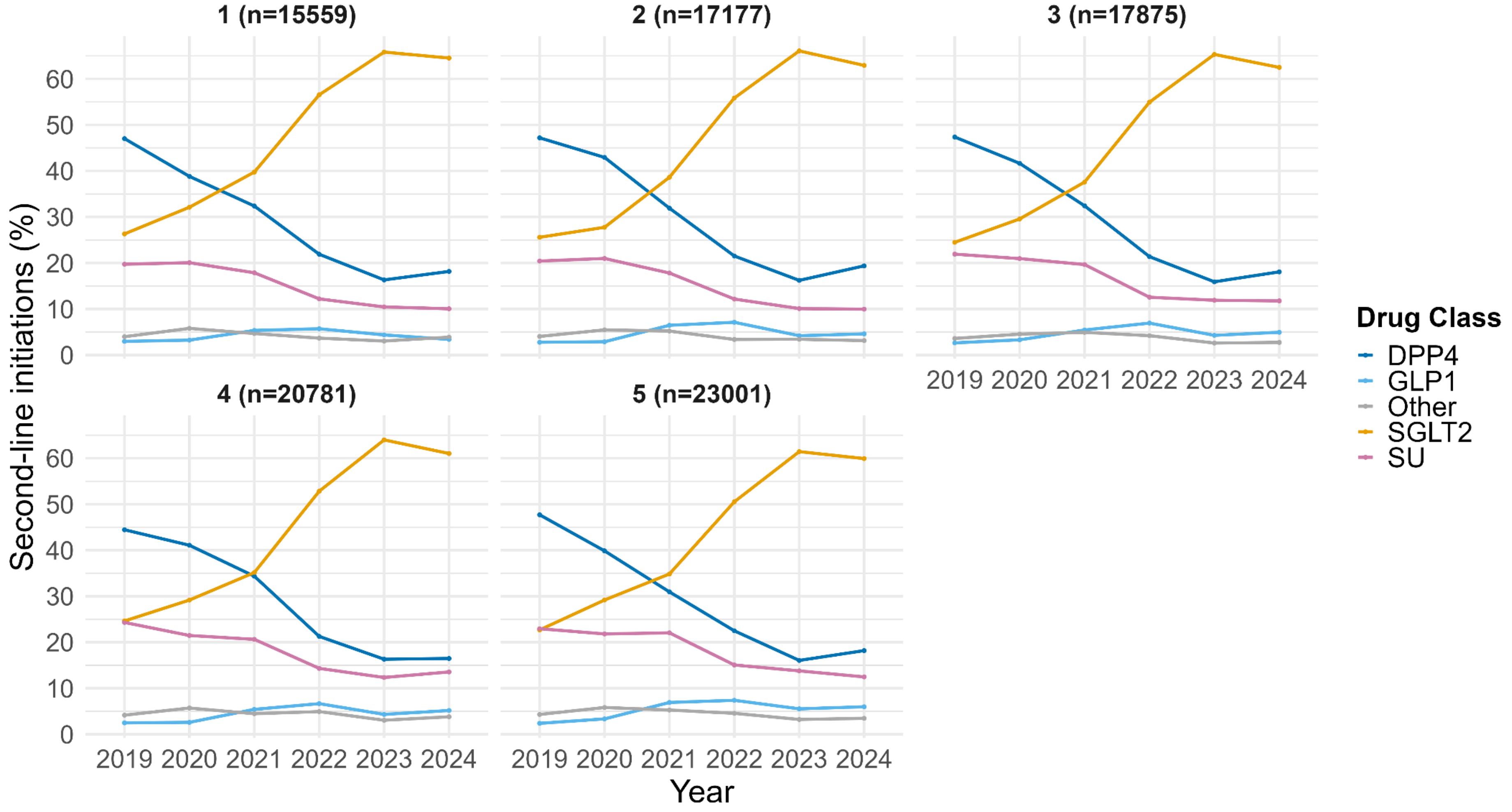

Supplementary Figure 4. Trends in second-line initiations by baseline cardiovascular disease status (2019-2024).

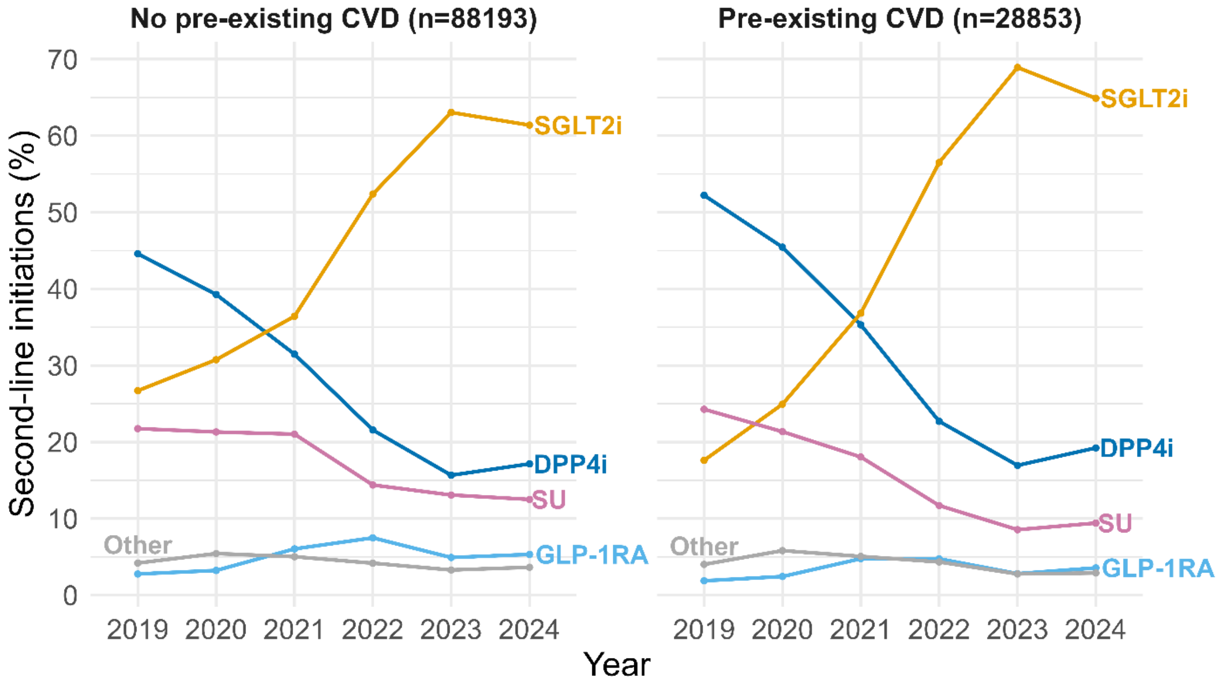

Supplementary Figure 5. Trends in second-line initiations by baseline chronic kidney disease status (2019-2024).

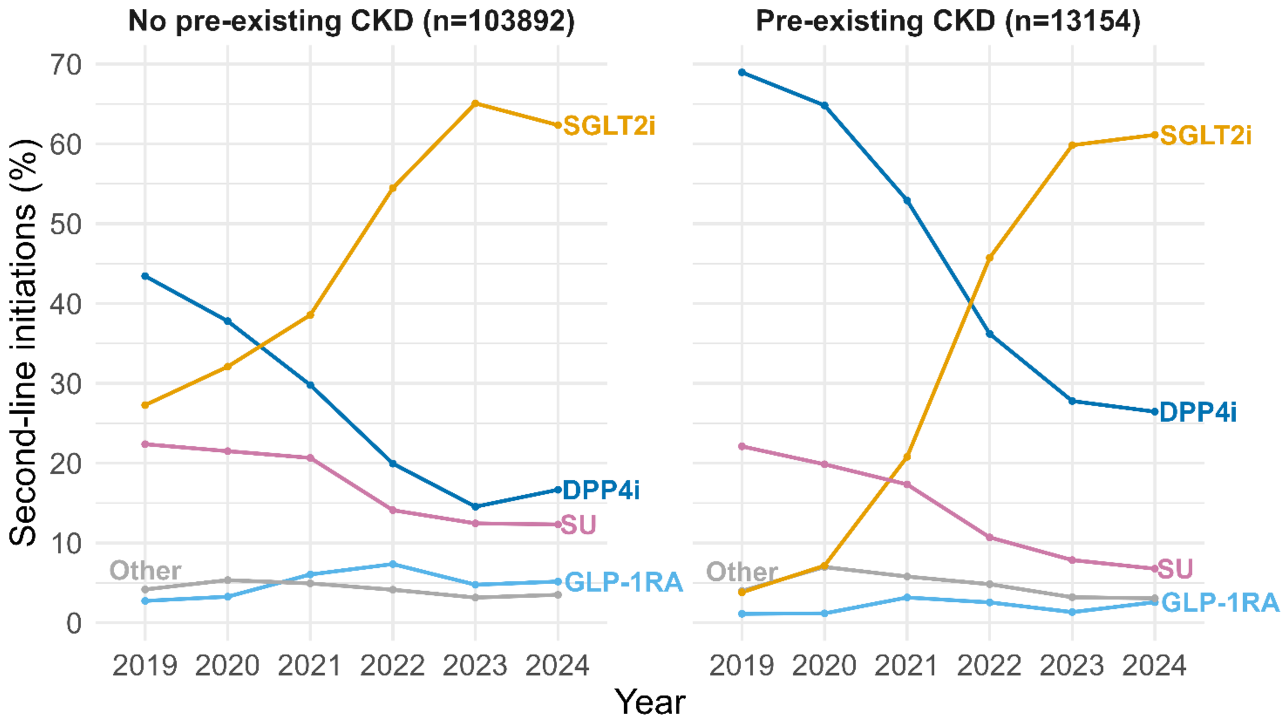

Supplementary Figure 6. Trends in second-line initiations by age and frailty subgroup (2019-2024), with frailty further categorised into moderate and severe.

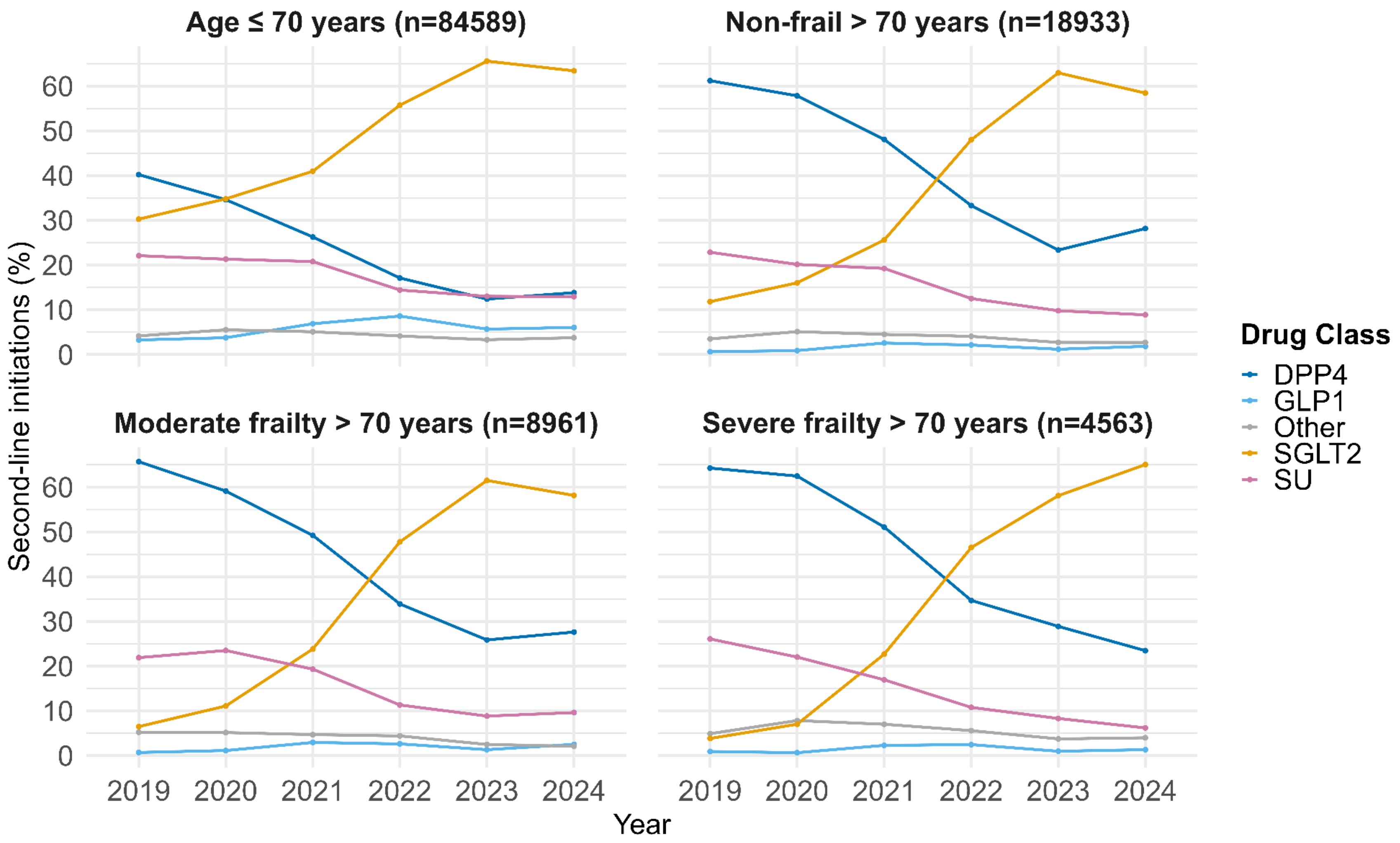

Supplementary Figure 7. 6-month HbA1c response, weight change (2019-2023) and treatment discontinuation (2019-2022) following second-line therapy initiation by age and frailty subgroups.

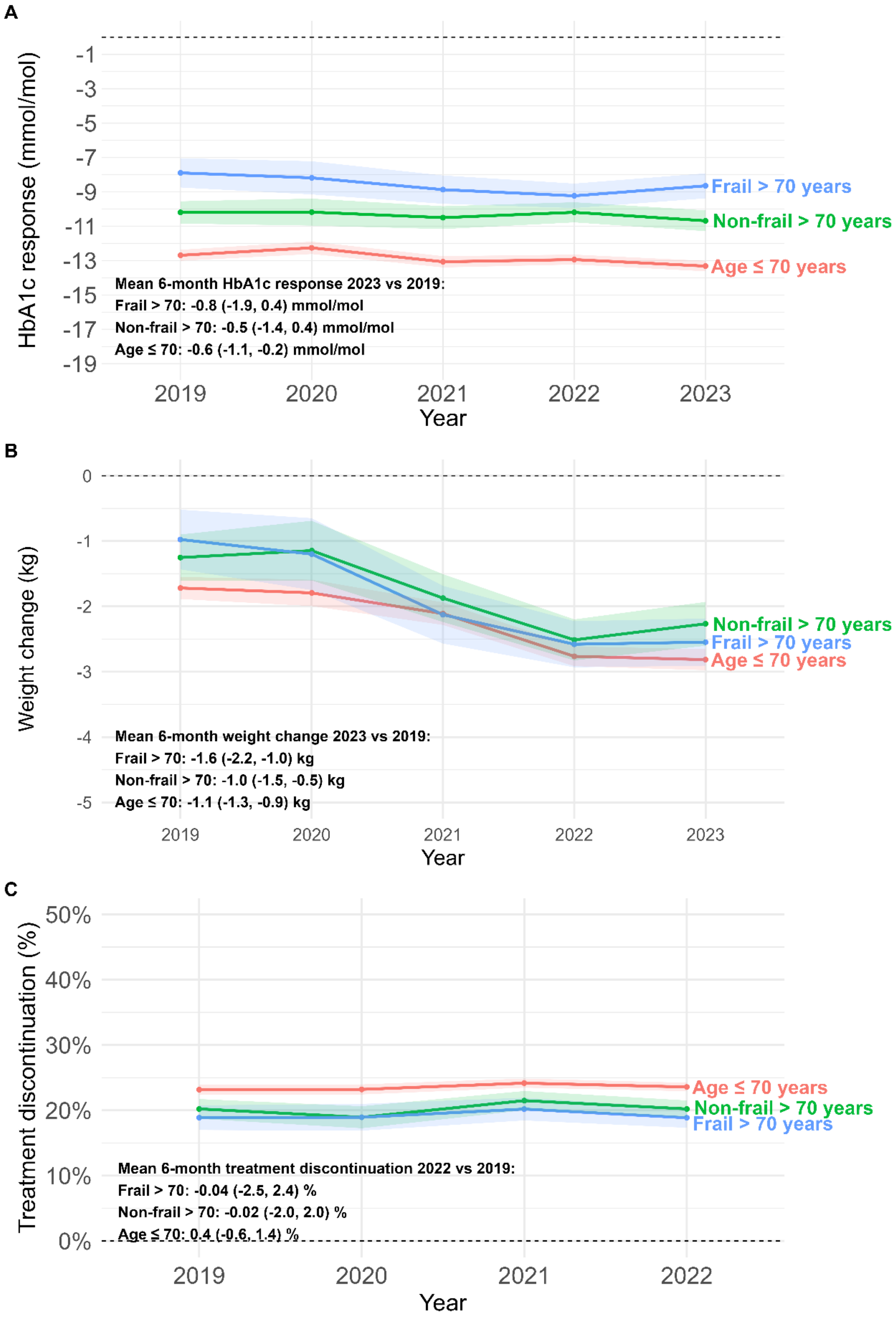

Supplementary Figure 8. 12-month HbA1c response, weight change (2019-2023) and treatment discontinuation (2019-2022) following second-line therapy initiation by age and frailty subgroups, with frailty further categorised into moderate and severe.

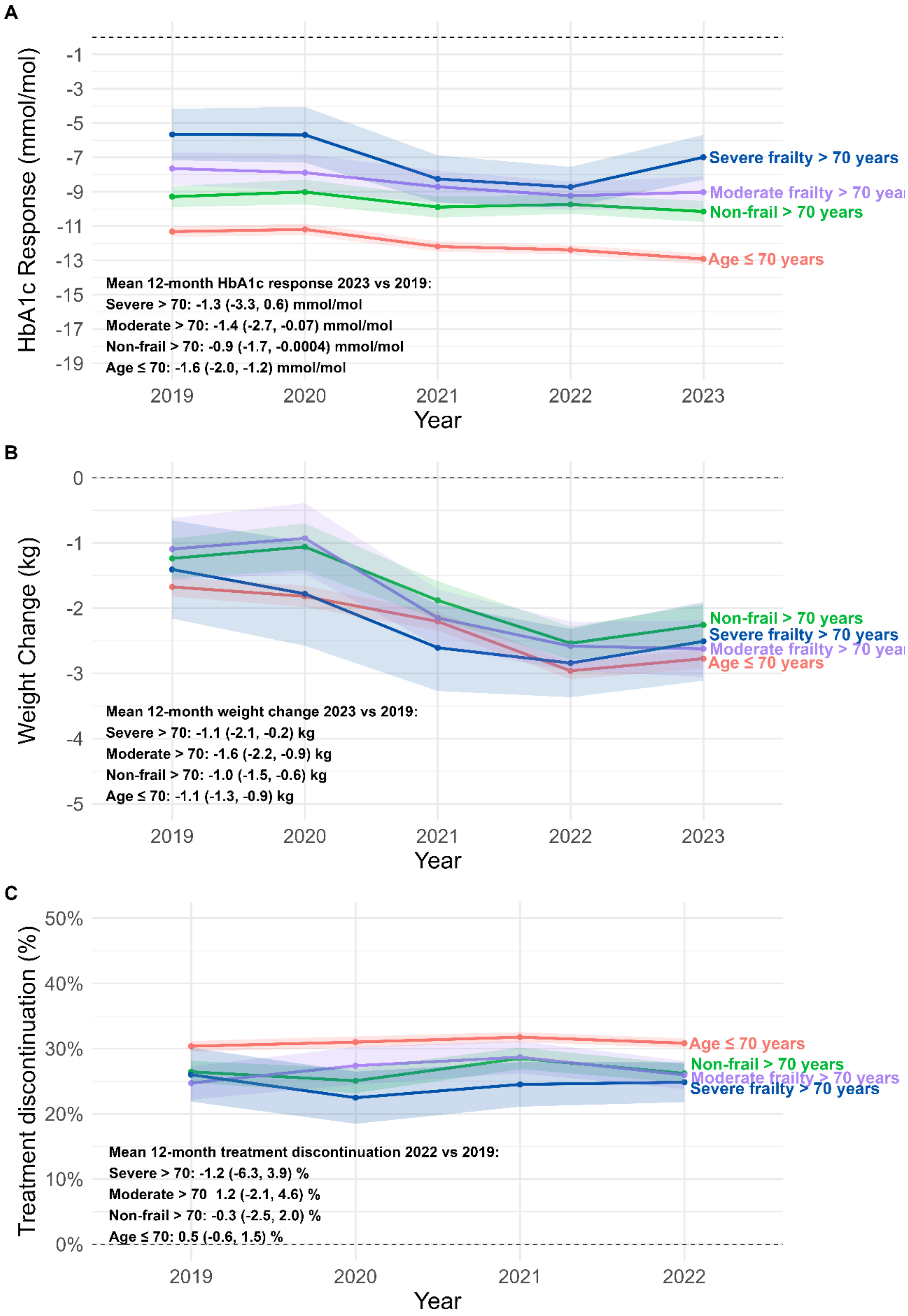

Supplementary Table 3. Crude incidence rates of complications following second-line therapy initiation including a severe diabetes-related complication, heart failure, kidney failure and DKA, per 1000 person-years by calendar year (2019–2022) and frailty category a) age ≤ 70 (n= 84,589) b) non-frail > 70 (n=18,933) c) frail > 70 (n=13,524).

a) Age ≤ 70 years

Outcome	Year	n	No. of outcome events	Crude incidence rate /1000 person-years
**Severe diabetes-related complications[a]**	2019	11367	111	12.9
	2020	9892	99	13.3
	2021	12327	117	13.0
	2022	14153	99	13.0
**Heart failure[b]**	2019	11367	20	2.3
	2020	9892	25	3.4
	2021	12327	35	3.9
	2022	14153	33	4.3
**Kidney failure[c]**	2019	11367	20	2.3
	2020	9892	18	2.4
	2021	12327	12	1.3
	2022	14153	14	1.8
**DKA**	2019	11367	5	0.6
	2020	9892	8	1.1
	2021	12327	11	1.2
	2022	14153	5	0.7

b) Non-frail > 70 years

Outcome	Year	n	No. of outcome events	Crude incidence rate /1000 person-years
**Severe diabetes-related complications[a]**	2019	2613	38	19.0
	2020	1904	28	19.2
	2021	2728	51	25.4
	2022	3279	48	27.0
**Heart failure[b]**	2019	2613	11	5.5
	2020	1904	8	5.4
	2021	2728	24	11.9
	2022	3279	17	9.5
**Kidney failure[c]**	2019	2613	6	3.0
	2020	1904	3	2.0
	2021	2728	2	1.0
	2022	3279	4	2.2
**DKA**	2019	2613	n < 5	0.0
	2020	1904	n < 5	0.7
	2021	2728	n < 5	1.0
	2022	3279	n < 5	1.1

c) Frail > 70 years

Outcome	Year	n	No. of outcome events	Crude incidence rate /1000 person-years
**Severe diabetes-related complications[a]**	2019	1715	80	64.2
	2020	1436	69	68.4
	2021	1972	87	62.1
	2022	2416	106	82.8
**Heart failure[b]**	2019	1715	39	30.9
	2020	1436	29	28.3
	2021	1972	54	38.1
	2022	2416	61	47.2
**Kidney failure[c]**	2019	1715	7	5.5
	2020	1436	9	8.7
	2021	1972	8	5.6
	2022	2416	5	3.8
**DKA**	2019	1715	n < 5	0.0
	2020	1436	n < 5	0.0
	2021	1972	6	4.2
	2022	2416	n < 5	0.8

[a] Severe diabetes-related complication: sudden death, death from hyperglycaemia or hypoglycaemia, fatal or non-fatal myocardial infarction, angina, fatal or non-fatal heart failure, fatal or non-fatal stroke, fatal or non-fatal kidney failure, death from peripheral vascular disease, amputation, blindness and severe retinopathy (vitreous haemorrhage, retinal photocoagulation)

[b]Heart failure: Fatal or non-fatal heart failure

[c]Kidney failure: Fatal or non-fatal kidney failure

Supplementary Figure 9. Rate (per 1000 person-years) following second-line therapy initiation of heart failure hospitalisations in patients with pre-existing heart failure or cardiovascular disease across age and frailty subgroups.

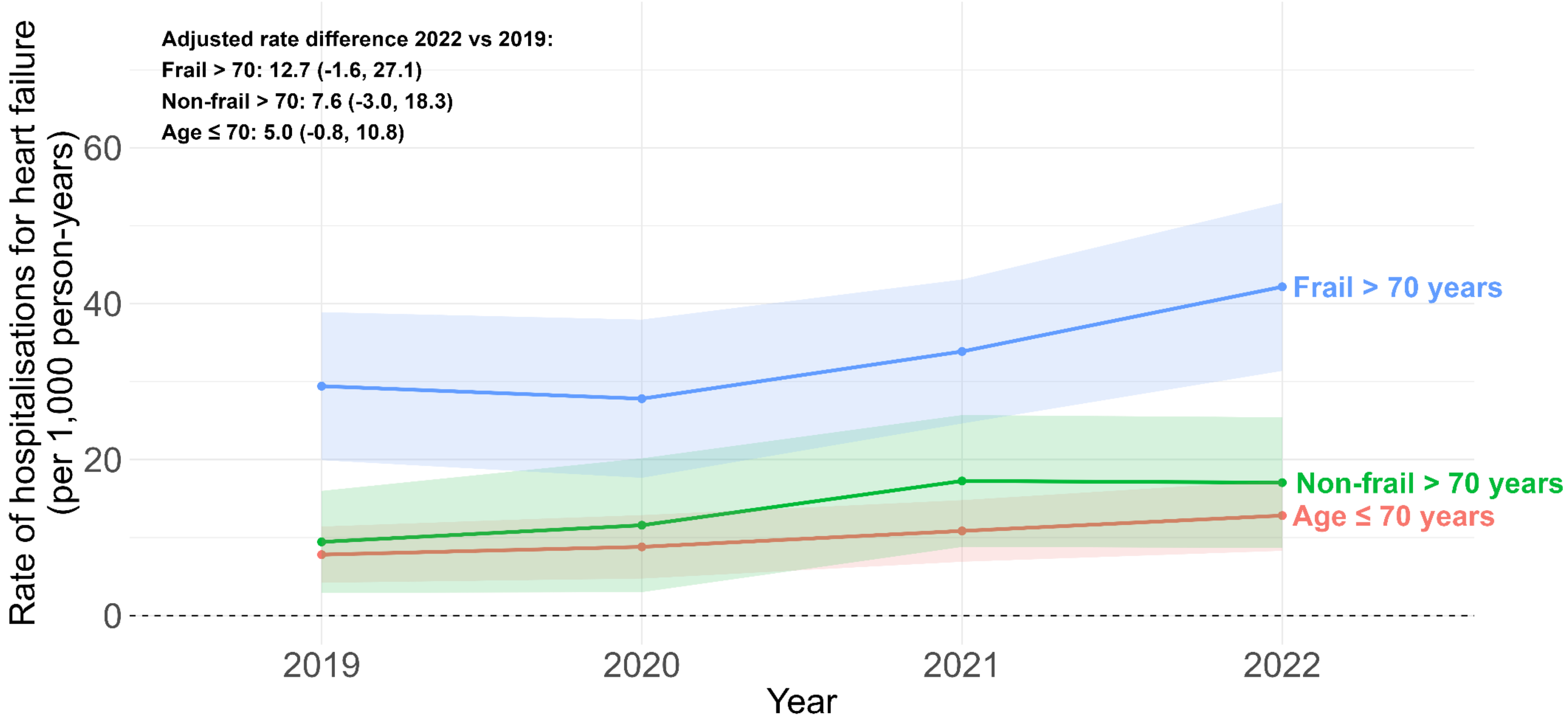

Supplementary Figure 10. Interrupted time series analysis of monthly SGLT2i initiations following 2022 NICE guideline update by age and frailty subgroups, 2019-2024. The solid black line shows the observed trend, the dashed red line shows the counterfactual trend, and the vertical dashed blue line shows the intervention (publication of the NICE guidelines).

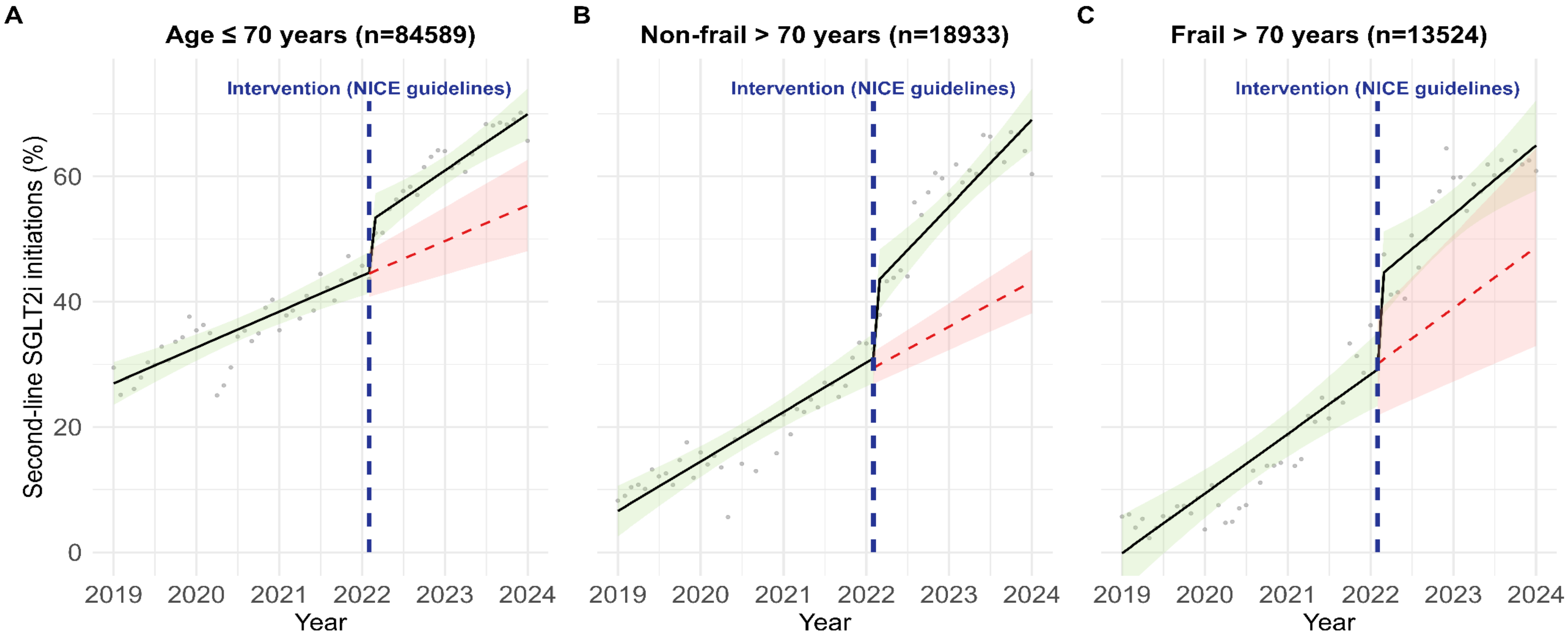

Supplement: Supplementary file 1 — Table S1: Baseline characteristics of the study cohort at second‐line treatment initiation by calendar year (2019–2024). Table S2: Baseline characteristics of the study cohort at second‐line treatment initiation with age and frailty subgroups further stratified into moderate and severe frailty. Table S3: Crude incidence rates of complications following second‐line therapy initiation including a severe diabetes‐related complication, heart failure, kidney failure and DKA, per 1000 person‐years by calendar year (2019–2022) and frailty category a) age ≤ 70 (n = 84 589) b) non‐frail > 70 (n = 18 933) c) frail > 70 (n = 13 524). Flowchart S1. CPRD patient flow and inclusion criteria for individuals initiating second‐line glucose‐lowering therapy and those included in each analysis. Figure S1: Trends in second‐line initiations by sex (2019–2024). Figure S2: Trends in second‐line initiations by ethnicity (2019–2024). Figure S3:. Trends in second‐line initiations by deprivation (IMD quintiles) (2019–2024). Figure S4: Trends in second‐line initiations by baseline cardiovascular disease status (2019–2024). Figure S5: Trends in second‐line initiations by baseline chronic kidney disease status (2019–2024). Figure S6: Trends in second‐line initiations by age and frailty subgroup (2019–2024), with frailty further categorised into moderate and severe. Figure S7: 6‐month HbA1c response, weight change (2019–2023) and treatment discontinuation (2019–2022) following second‐line therapy initiation by age and frailty subgroups. Figure S8: 12‐month HbA1c response, weight change (2019–2023) and treatment discontinuation (2019–2022) following second‐line therapy initiation by age and frailty subgroups, with frailty further categorised into moderate and severe. Figure S9: Rate (per 1000 person‐years) following second‐line therapy initiation of heart failure hospitalisations in patients with pre‐existing heart failure or cardiovascular disease across age and frailty subgroups. Figure S10: Int [file DOM-28-7471-s001.pdf]
